# Characteristics, Motivations, and Preferences of Healthy Volunteers in Phase I Clinical Trials in Sweden

**DOI:** 10.1177/15562646241309142

**Published:** 2025-01-27

**Authors:** Erik Rein-Hedin, Mårten Schultzberg, Folke Sjöberg, Fredrik Huss

**Affiliations:** 1 690308 CTC Clinical Trial Consultants AB, Uppsala, Sweden; 2 Department of Surgical Sciences, Plastic Surgery, 8097 Uppsala University, Uppsala, Sweden; 3 Department of Statistics, 8097 Uppsala University, Uppsala, Sweden; 4 Department of Biomedical and Clinical Sciences, 4566 Linköping University, Linköping, Sweden

**Keywords:** healthy volunteers, clinical trials, risks, benefits, and burdens of research/beneficence and non-maleficence, research ethics, survey research

## Abstract

This study examined the characteristics, experiences, motivations, and preferences of healthy volunteers participating in Phase I clinical trials in Sweden. A descriptive, cross-sectional survey was conducted among 135 healthy volunteers who had participated in at least one Phase I clinical trial from 2021 to 2024. Volunteers considered trial personnel, financial compensation, and regulatory review and approval as highly important factors in their decision to participate. Willingness to participate varied depending on trial characteristics, with greater reluctance for trials involving discomfort or perceived higher risks. Experiences, motivations, and preferences were comparable irrespective of age, gender, occupation, or income. Participants had disproportionately low incomes but reported financial satisfaction comparable to the general population. Unlike findings in other regions, there was no overrepresentation of migrants or the unemployed. Insights from this study can help guide trial design considerations to facilitate equitable recruitment and reduce the burden of participation.

## Background/Aims

Recruiting healthy volunteers for clinical trials presents ethical challenges with respect to ensuring informed consent, fair compensation, and minimizing risk. Phase I trials, including first-in-human trials, are often conducted in healthy volunteers. These trials are not limited to the first safety and tolerability studies but also include pharmacokinetic trials evaluating bioavailability, drug-drug interaction and metabolism (Bompart et al., 2023). Testing in healthy individuals reduces the potential for confounding factors that can affect interpretation and trial results. Furthermore, healthy volunteers are usually able to better tolerate unforeseen adverse events compared to patients with pre-existing conditions (Dresser, 2009).

Recruiting subjects for clinical trials is a frequent bottleneck that affects trial timelines and causes delays (Treweek et al., 2018). While this is specifically true for patient trials with narrow inclusion and exclusion criteria, recruiting healthy, willing, and suitable volunteers can also pose challenges. For example, in a French online survey during the COVID-19 pandemic, 75% of respondents were willing to take the COVID-19 vaccine but only 48% were willing to participate in a COVID-19 vaccine trial (Detoc et al., 2020).

Participation in clinical trials is always voluntary, and subjects may withdraw from participation at any time. It is consequently equally important to enroll subjects motivated to not just enter but also stay in the trial, and comply with study procedures, restrictions, and requirements. Several efforts, such as EUPATI and CTII, have been established to enhance patient engagement and involvement in the drug development process, including clinical trial design (CTTI, n.d.; EUPATI, 2023). In cancer research, there has since 2021 been a political goal in the EU to actively involve patients in trial design (German Federal Ministry of Education and Research, n.d.). The pharmaceutical industry has similarly demonstrated increased awareness of a patient-centric approach where patient needs and lifestyles are given consideration (Faulkner et al., 2023; Sharma, 2015).

Substantially less attention has been given to the healthy volunteer, though there are now recent international efforts focused on discussing ethical issues and best practices regarding healthy volunteers, such as the VolREthics (Volunteers in Research and Ethics) Initiative (Bompart et al., 2023). Healthy volunteers in clinical trials are exposed to risks and burdens, without any potential benefit from the investigational drug (vaccines excepted). From an ethical perspective, not only risk but also burden of participation should wherever possible be minimized for all subjects in clinical trials. For early trials with relatively few participants, voluntary withdrawal of even a few subjects can also have a substantial impact on evaluability of study data, which undermines the efforts of all participants. Even if financial compensation is generally considered the main motivation, (Almeida et al., 2007; Grady et al., 2017; Lemmens & Elliott, 1999; Stunkel & Grady, 2011) it should be noted that healthy volunteers will typically be financially compensated only for time spent, efforts and burdensome procedures during the trial. According to Good Clinical Practice (GCP), FDA guidance, EU legislation, and other applicable and regional ethical standards, no undue influence, including that of a financial nature, must be exerted on subjects to participate in a clinical trial (European Medicines Agency, 2016; Regulation (EU) No 536/2014, 2022; U.S. Food and Drug Administration, 2019). Compensation should be ethically justified and must not be coercive or unduly influential, that is, offer of significant payment must not make a volunteer participate when they otherwise would not (Largent et al., 2012). On the other hand, while Institutional Review Boards and Independent Ethical Committees generally take a conservative approach toward paying research participants, it has been argued that the risk of high compensation being unduly influential is limited, and that the risk of exploitation due to compensation being too low may be of greater concern (Lamkin & Elliott, 2018; Largent & Fernandez Lynch, 2017).

Clinical research should be inclusive and represent people of different genders, races, ethnicities, ages, and socioeconomic statuses. This particularly applies to later-stage trials, as exemplified by the recent FDA draft guidance, requiring a Diversity Action Plan for Phase III and pivotal studies (U.S. Food and Drug Administration, 2024). However, it has been argued that diversity also in early trials with healthy volunteers holds several benefits, such as increased scientific robustness and public trust (Chaudhry et al., 2022). In the US, racial and ethnic minorities, as well as disadvantaged groups, have been found to be overrepresented in healthy volunteer Phase I trials. Since healthy participants are exposed to risk and burden without potential therapeutic benefit, this has been argued to cause a disproportionate burden to these groups. The same groups often lack representation in later-stage trials with potential therapeutic gains (Kalbaugh et al., 2021). Women, as well as gender minorities, are also underrepresented in Phase I trials and medical research in general, which might negatively affect the ability to recognize differences in safety and pharmacokinetics during the early stages of drug development (Burgwal et al., 2019; Chen et al., 2018; Kalbaugh et al., 2021). Depending on the therapeutic area, drug, and study design, there might additionally be rationale for recruiting healthy volunteers with specific characteristics, including but not limited to, gender, genetic variants and age.

Better understanding of subjects’ motivations and decision-making process is necessary to facilitate diverse and successful recruitment of healthy volunteers. Factors that encourage or discourage participation should be further examined to improve trial experience, increase representation, and better trial design to reduce burden where possible. The current research remains limited. Much of the published data today on healthy volunteers is over a decade old and may not reflect the current younger generation and potential shifts in attitudes. Considering regional differences, it is necessary to conduct studies across all areas where trials are conducted.

The aims of this study are to examine demographic characteristics of healthy volunteers in Phase I trials in Sweden, key motivations, and decision-influencing factors, as well as to determine potential relationships between prior experiences, demographic characteristics, and motivations. To our knowledge, no similar research studying healthy volunteers has previously been conducted in Sweden or the Nordic countries.

## Methods

### Study Design

This descriptive, cross-sectional survey study included healthy volunteers aged 18 or older who had participated in at least one Phase I clinical pharmaceutical trial as healthy volunteers. The STROBE cross-sectional reporting guidelines were used (von Elm et al., 2008). Pharmaceutical trials were defined as trials where all volunteers were administered an investigational medicinal drug (IMP), or IMP or placebo in a blinded fashion. Participants were asked to take the survey after the last visit in their respective trial. The purpose of collecting survey data after trial completion was to ensure all respondents had completed participation in at least one Phase I trial and were not currently enrolled in a trial at the time of responding. Completed participation was defined as having been enrolled, administered IMP or placebo, and attended the final follow-up visit, or if applicable attended a scheduled early withdrawal visit replacing the final follow-up visit. Any voluntary dropouts during a trial who did not wish to return for a scheduled visit could not be approached. Each volunteer was only eligible to take the survey once, even if participating in more than one trial during the data collection period. The study was approved by the Swedish Ethical Review Authority (Dnr 2021-01068) and was conducted at two research units run by CTC Clinical Trial Consultants AB in Uppsala, Sweden. All participants were given verbal and written information about the study, including background and purpose, had the opportunity to ask questions and provided written informed consent before participation. Study information included that participants were free to skip questions at their own discretion. Participants were assured that responses would remain confidential and that their decision to participate would not affect their relationship with the trial site or eligibility for future studies.

The survey was developed through a stepwise process that included literature review, draft survey development, review by clinical trial professionals and statistician, revisions, pretesting with 20 Phase I healthy volunteers, and final revisions. Survey questions covered four domains: demographic characteristics, previous experience from clinical trials, decision-influencing factors for participation, and willingness to participate in hypothetical trials. Respondents rated statements and questions on a five-point Likert scale ranging from “not at all” to “very”, evaluating aspects such as likelihood, importance, sufficiency, and trust. The survey ended with a comments section, allowing participants to provide further feedback or elaborate on their responses. The survey was in Swedish language. An English translation of the survey is provided in Supplementary Material 1.

### Statistical Analysis

Data were obtained either through paper or electronic forms (Microsoft Forms, Microsoft Corporation, Redmond, WA). Pseudonymized data were entered or exported to Microsoft Excel (Microsoft Corporation, Redmond, WA). Statistical calculations were performed using RStudio 2024.04.2 (Posit Software, PBC, Boston, MA) using packages “Tidyverse” (Wickham et al., 2019), “Hmisc” (Harrell, 2024) and “dunn.test” (Dinno, 2024).

Frequency distributions and descriptive statistics were used to describe the data. 95% confidence intervals (CIs) for proportions were calculated using the exact method, (Clopper & Pearson, 1934) which was used consistently regardless of proportion size. Bivariate correlation between ordinal data (Likert scale data and ranked background variables), as well as age, was measured using non-parametric tests, i.e., Spearman rank correlation (Best & Roberts, 1975). Required sample size was determined based on the Fisher z-transform of the Spearman correlation coefficient. A sample size of 85 participants was determined sufficient to achieve 80% power for a two-tailed test of a single Spearman coefficient at significance level 0.05, in order to detect at least moderate correlation (0.3) between variables (May & Looney, 2020).

Kruskal-Wallis test (Kruskal & Wallis, 1952) was used to compare ordinal data, including age, across multiple categorical groups (marital status, occupation). For binary categorical data, Mann-Whitney U test was used (Mann & Whitney, 1947). To correct for multiple comparisons across all tests and reduce the risk of false positives, a global *p*-value adjustment was performed using the Hochberg method (Hochberg, 1988). For significant Kruskal-Wallis results, post-hoc analysis was conducted using Dunn's test (Dunn, 1964) and without additional *p*-value adjustment. For missing data, analyses were conducted with available data, and each variable reflect the number of responses. No imputation was performed for missing values, as the frequency of missing data was below 3% for all variables.

## Results

### Background Characteristics

Out of 158 healthy volunteers invited to take the survey between April 2021 and February 2024, a total of 135 (85%) participated. Nonparticipation was primarily due to participants forgetting to take the survey after indicating interest or being in a hurry to leave after their last study visit due to other obligations. Background characteristics are summarized in Table 1. The mean age was 35.3 years (SD 11.5). A substantial majority (89%; CI 82%–93%) were Swedish-born and approximately half (56%; CI 47%–64%) were male. All participants identified as being either male or female. About half (51%; CI 41%–60%) were single and a majority (73%; CI 65%–81%) had no children living at home. Half (50%; CI 41%–58%) were employed, approximately one-third (36%; CI 28%–44%) students and one-tenth (9%; CI 5%–15%) reported being unemployed (i.e., active job seekers and available for work). Two-thirds (67%; CI 59%–75%) had higher education defined as studies and/or a degree at university/college level. A majority (73%; CI 65%–81%) had a personal monthly income of less than 30,000 Swedish krona (SEK), and about half (58%; CI 49%–66%) less than 20,000 SEK. Half (51%; CI 42%–60%) of the participants took this survey after having participated in a clinical trial for the first time.

**Table 1.** Background Characteristics.

		Mean	SD	95% CI
Age (years) (n = 135)		35.3	11.5	33.4–37.3
		**n**	**%**	**95% CI**
Gender (n = 135)				
	Female	60	44.4%	35.9%–53.2%
	Male	75	55.6%	46.8%–64.1%
Country of birth (n = 135)				
	Sweden	120	88.9%	82.3%–93.4%
	Other country	15	11.1%	6.4%–17.7%
Marital status (n = 134)				
	Single	68	50.7%	41.2%–59.5%
	Married	26	19.4%	13.1%–27.1%
	Domestic partnership	28	20.9%	14.4%–28.8%
	Living apart together	8	6.0%	2.6%–11.4%
	Divorced/separated	3	2.2%	0.5%–6.4%
	Widow/widower	1	0.7%	0.0%–4.1%
Children at home (n = 135)				
	Yes	36	26.7%	19.4%–35.0%
	No	99	73.3%	65.0%–80.6%
Education (n = 135)				
	Primary education	3	2.2%	0.5%–6.4%
	Upper secondary education	26	19.3%	13.0%–26.9%
	Post-secondary non-tertiary education	15	11.1%	6.4%–17.7%
	Higher education at university/college level	34	25.2%	18.1%–33.4%
	University/college degree	57	42.2%	33.8%–51.0%
Occupation (n = 135)				
	Employed	67	49.6%	40.9%–58.4%
	Unemployed	12	8.9%	4.7%–15.0%
	Parental leave	2	1.5%	0.2%–5.3%
	Homemaker	2	1.5%	0.2%–5.3%
	Student	48	35.6%	27.5%–44.3%
	Long-term sick leave/early retirement	1	0.7%	0.0%–4.1%
	Retired	3	2.2%	0.5%–6.4%
Travel distance to clinic (km) (n = 135)				
	0–9	59	43.7%	35.2%–52.5%
	10–19	10	7.4%	3.6%–13.2%
	20–49	15	11.1%	6.4%–17.7%
	50–99	39	28.9%	21.4%–37.3%
	≥ 100	12	8.9%	4.7%–15.0%
Monthly income (SEK) (n = 135)				
	0–9,999	24	17.8%	11.7%–25.3%
	10–19,999	54	40.0%	31.2%–48.8%
	20–29,999	21	15.6%	9.9%–22.8%
	30–39,999	26	19.3%	13.0%–26.9%
	40–49,999	6	4.4%	1.7%–9.4%
	50–59,999	2	1.5%	0.2%–5.3%
	60–69,999	2	1.5%	0.2%–5.3%
Financial satisfaction (n = 135)				
	Very satisfied	11	8.1%	4.1–14.1%
	Fairly satisfied	40	29.6%	22.1%–38.1%
	Neither satisfied nor dissatisfied	41	30.4%	22.8%–38.9%
	Fairly dissatisfied	34	25.2%	18.1%–33.4%
	Very dissatisfied	9	6.7%	3.1%–12.3%
Self-perceived health (n = 135)				
	Very good	81	60.0%	51.2%–68.3%
	Fairly good	51	37.8%	29.6%–46.5%
	Neither good nor bad	3	2.2%	0.5%–6.4%
Previous trials (n = 135)				
	1	69	51.1%	42.4%–59.8%
	2	31	23.0%	16.2%–31.0%
	3	8	5.9%	2.6%–11.3%
	4	10	7.4%	3.6%–13.2%
	≥ 5	17	12.6%	7.5%–19.4%

CI = confidence interval; n = number of subjects; SD = Standard Deviation; SEK = Swedish krona.

### Previous Experiences

Detailed means and standard deviations for previous experience items are presented in Table 2. A significant majority expressed a high likelihood of recommending trial participation to friends or family, with 87% (CI 80%–92%) responding they were fairly or very likely to do so. Financial compensation was deemed adequate by most (57%; CI 48%–66%). Volunteers reported high satisfaction with the verbal and written trial information, with 97% (CI 93%–99%) considering the information sufficient or more than sufficient. Trust in the competence of trial personnel was particularly high, with 99% (CI 95%–100%) expressing fairly or very high trust. Trust in the reviewing authorities (the Swedish Medical Products Agency and the Swedish Ethical Review Authority) was also high, with 91% (CI 85%–95%) expressing fairly or very high trust. Worry about side effects averaged a low score, with 3% (CI 1%–8%) reporting having been fairly or very worried. The challenge of participation compared to expectations received a moderate score, with about one-fourth (27%; CI 19%–35%) finding it more challenging than anticipated. Reactions from relatives and close friends towards their participation were mixed, with about half (47%; CI 38%–56%) noting positive reactions. Notably, one in four (26%; CI 19%–34%) reported receiving advice against participation from family or friends, with concerns including the risk of side effects (25%; CI 18%–33%), inadequate compensation (7%; CI 3%–12%), and missing time with family or social gatherings (4%; CI 1%–8%).

**Table 2.** Previous Experiences.

Question (n)	mean	SD		95% CI
			Percentage fairly/very high trust	
Trust in the competence of trial personnel (134)	4.81	0.43	98.5%	94.7%–99.8%
(1 = Very low trust; 5 = Very high trust)				
			Percentage sufficient/more than sufficient	
Thoughts on verbal and written trial information received (135)	4.41	0.58	97.0%	92.6%–99.2%
(1 = Not at all sufficient; 5 = More than sufficient)				
			Percentage fairly/very high trust	
Trust in authorities^a^ that reviewed and approved the trial? (135)	4.41	0.77	91.1%	85.0%–95.3%
(1 = Very low trust; 5 = Very high trust)				
			Percentage fairly/very likely	
Likely to participate in more trials (133)	4.38	0.71	89.5%	83.0%–94.1%
(1 = Not at all likely; 5 = Very likely)				
			Percentage fairly/very likely	
Likely to recommend a friend/family member to participate in a trial? (134)	4.37	0.72	87.3%	80.4%–92.4%
(1 = Not at all likely; 5 = Very likely)				
			Percentage sufficient/more than sufficient	
Thoughts on financial compensation (135)	3.39	0.94	57.0%	48.2%–65.5%
(1 = Not at all sufficient; 5 = More than sufficient)				
			Percentage fairly/very positive	
Reaction of relatives and close friends to participation? (133)	3.43	0.91	46.6%	37.9%–55.5%
(1 = Very negative; 5 = Very positive)				
			Percentage slightly/much more challenging	
Challenge of participation compared to expectations (135)	3.10	0.82	26.7%	19.4%–35.0%
(1 = Not at all as challenging; 5 = Much more challenging)				
			Percentage fairly/very worried	
Worry about experiencing side effects (134)	1.86	0.78	3.0%	0.8%–7.5%
(1 = Not at all worried; 5 = Very worried)				
	Yes	No	Percentage yes	
Did you discuss your participation with anyone? (135)	104	31	77.0%	69.0%–83.8%
Discussed with, multiple choices possible	n			
Family member/partner	84		62.2%	53.5%–70.4%
Friend	53		39.3%	31.0%–48.0%
Healthcare professional	7		5.2%	2.1%–10.4%
Others	5		3.7%	1.2%–8.4%
	Yes	No	Percentage yes	
Has any family member/friend advised you against participating? (135)	35	100	25.9%	18.8%–34.2%
Reasons given, multiple choices possible	n			
Risk of side effects	34		25.2%	18.1%–33.4%
Too low compensation	9		6.7%	3.1%–12.3%
Missing time with family/social gatherings	5		3.7%	1.2%–8.4%
Other reasons	5		3.7%	1.2%–8.4%

a = Swedish Medical Products Agency and Swedish Ethical Review Authority; CI = confidence interval; n = number of subjects; SD = Standard Deviation.

### Decision-Influencing Factors

Factors that were considered fairly or very important for willingness to participate by more than 90% of participants were competence of the trial personnel (99%; CI 95%–100%), that the trial had been reviewed by authorities (96%; CI 92%–99%), respectful and considerate treatment from personnel (94%; CI 89%–97%), and the amount of financial compensation (94%; CI 89%–97%) (Table 3). In contrast, less than 50% considered learning about clinical trials (41%; CI 33%–50%), short trial duration (39%; CI 31%–48%), how the drug is administered (32%; CI 24%–40%), positive attitude of family and friends (26%; CI 19%–34%), and the opportunity to get to know new people (26%; CI 19%–34%), to be fairly or very important for their willingness to participate. These five factors showed relatively higher standard deviations (≥1.10, as compared to 0.47–1.08 for the remaining), indicating greater variability in responses.

**Table 3.** Decision-Influencing Factors.

Factor (n)	Mean	SD	Percentage fairly/very important	95% CI
(1 = Not at all important; 5 = Very important)				
Competence of trial personnel (physicians and nurses) (135)	4.74	0.47	98.5%	94.8%–99.8%
Trial reviewed and approved by authorities (135)	4.75	0.54	96.3%	91.6%–98.8%
Amount of financial compensation (135)	4.49	0.68	94.1%	88.7%–97.4%
Respectful/considerate treatment from trial personnel (134)	4.50	0.61	94.0%	88.6%–97.4%
Contribution to medical research (135)	4.26	0.72	86.7%	79.8%–91.9%
Comprehensive health check as part of the trial (135)	4.22	0.91	80.0%	72.3%–86.4%
Low likelihood of experiencing side effects (135)	4.03	0.91	76.3%	68.2%–83.2%
Standard of trial facilities (beds, common rooms, Wi-Fi, etc) (135)	3.83	0.95	71.1%	62.7%–78.6%
Flexibility to change visit days and times to fit my life (134)	3.78	0.96	67.2%	58.5%–75.0%
Drug indication (134)	3.70	1.08	61.2%	52.4%–69.5%
Learning about clinical trials (134)	3.06	1.10	41.0%	32.6%–49.9%
Short trial duration with few visits (135)	3.07	1.18	39.3%	31.0%–48.0%
How the drug is administered (135)	2.84	1.19	31.9%	24.1%–40.4%
Positive attitude of family and friends (135)	2.66	1.10	25.9%	18.8%–34.2%
Opportunity to get to know and socialize with new people (135)	2.76	1.14	25.9%	18.8%–34.2%

CI = confidence interval; n = number of subjects; SD = Standard Deviation.

### Willingness to Participate in Hypothetical Trials

Participants were asked to consider their willingness to participate in hypothetical trials with defined characteristics (Table 4). The highest willingness was reported for a trial requiring bedridden periods, with 81% (CI 74%–88%) reported being fairly or very likely to be willing to participate. The majority (69%; CI 60%–76%) were likely to participate in a trial where temporary, mild side effects could be expected, whereas only one-third (35%; CI 27%–44%) were willing to join a trial requiring undergoing an uncomfortable procedure (e.g., gastric tube insertion or lumbar puncture). Most participants were likely willing to take part in a trial investigating a new vaccine (64%; CI 55%–72%), whereas less than half (40%; CI 32%–49%) were likely to be willing to enroll in a trial where there have been serious side effects in animal studies at much higher doses than those used in the trial. One-third (34%; CI 26%–42%) reported being fairly or very likely to participate in a trial that has been reviewed and approved only by authorities in another EU country. Only 8% (CI 4%–14%) were likely to join a trial not approved by any authority. Trials with no financial compensation saw similarly low interest, with just 7% (CI 3%–12%) of participants fairly or very likely willing to participate.

**Table 4.** Willingness to Join Hypothetical Trials.

Type of trial (n)	Mean	SD	Percentage fairly/very likely	95% CI
(1 = Not at all likely; 5 = Very likely)				
A trial where you would need to be bedridden for parts of certain days due to frequent blood tests (134)	4.13	0.95	81.3%	73.7%–87.6%
A trial where the sponsor is a large, foreign, well-known company (134)	3.96	0.76	70.1%	61.6%–77.7%
A trial where temporary, mild side effects can be expected (134)	3.74	0.84	68.7%	60.1%–76.4%
A trial where the drug is a new vaccine (134)	3.72	0.91	64.2%	55.4%–72.3%
A trial where the sponsor is a small, unknown, Swedish company (134)	3.69	0.92	59.0%	50.1%–67.4%
A trial with long visits where you need to stay many nights (134)	3.60	1.11	55.2%	46.4%–63.8%
A first-in-human trial (134)	3.53	1.06	53.7%	44.9%–62.4%
A trial with frequent visits requiring many trips to and from the clinic (132)	3.34	1.06	49.2%	40.4%–58.1%
A trial where the medication is intended to affect the brain (134)	3.31	1.10	47.0%	38.3%–55.8%
A trial conducted at a clinic far from my home (134)	3.29	1.27	44.8%	36.2%–53.6%
A trial where serious side effects have been observed in animal studies at much higher doses than those used in the trial (134)	3.11	1.14	40.3%	31.9%–49.1%
A trial with an uncomfortable procedure (134)	2.87	1.19	35.1%	27.0%–43.8%
A trial that has been reviewed and approved only by authorities in another EU country (134)	2.96	1.08	33.6%	25.7%–42.3%
A trial that has not been reviewed or approved by any authority (134)	1.90	0.99	8.2%	4.2%–14.2%
A trial where you receive no financial compensation (134)	1.69	0.92	6.7%	3.1%–12.4%

CI = confidence interval; n = number of subjects; SD = Standard Deviation.

### Correlations and Associations Among Key Variables

Out of 1,035 bivariate correlations, 69 were significant (*p* < 0.05) after correction. These correlations are visualized in a heatmap (Supplementary Material 2).

A full summary for all tests is provided in Supplementary Material 3. The only correlation between background characteristics (as listed in Table 1) and previous experiences, decision-influencing factors and willingness to participate in hypothetical trials, was the expected correlation that participants living further from the trial site were more willing to participate in a trial conducted far from their homes (r = 0.58, *p* < 0.001). No other correlations or associations were found between background characteristics and previous experiences, decision-influencing factors, or willingness to participate in hypothetical trials.

### Comments and Feedback

Written comments and feedback from participants, with personal identifiable information redacted, are provided in Supplementary Material 4.

## Discussion

This study is, to our knowledge, the first to examine demographics, experiences, motivations, and enrollment willingness and preferences among healthy volunteers participating in clinical trials in Sweden and the Nordic countries.

### Demographics of Swedish Healthy Volunteers

Overall, the demographic profile in this study was largely consistent with studies from other regions. The average age (35.3) reflects the predominance of younger participants in healthy volunteer trials (Grady et al., 2017; Kalbaugh et al., 2021). While there is no authority guidance on what constitutes a “healthy volunteer”, (Deiteren et al., 2021) the age distribution can in part be explained by the upper age limit typically enforced in Phase I trials, difference in time constraints and responsibilities, and the increased prevalence of exclusionary co-morbidities and concomitant medications with increased age. Males made up just about half of the participants (56%; CI 47%–64%). Women and persons of childbearing potential can, and should whenever possible, be appropriately represented in early clinical trials. Guidelines do however require a short duration trial design with intensive control of pregnancy risk for their inclusion, which might be conservatively interpreted by sponsors and not considered achievable for all trials (European Medicines Agency, 2013; Jain et al., 2020).

Migrants may be considered a disadvantaged group, and such groups have been found to be overrepresented in Phase I trials (Kalbaugh et al., 2021). In this study, foreign-born participants were underrepresented at 11% (CI 6%–18%) compared with 21% in the general population in 2023, (Statistics Sweden, n.d.). This underrepresentation is even more pronounced considering the well-represented age group 25–45, where 31% of the general population in Sweden is foreign-born (Statistics Sweden, n.d.). For recent immigrants, this might be due to several reasons including language barriers, cultural differences and lack of awareness. It is worth mentioning that data on race and ethnicity were not collected in this study, as such data are considered sensitive personal data in Sweden, and ethical approval for its collection is only granted when it is deemed vital to the research objectives and the scientific value outweighs the intrusion into personal privacy.

Participants in this study were well-educated, with 67% (CI 59%–75%) having completed at least some college/university level education. The education level aligns with research from the US, Belgium, Singapore and South Korea, but contrasts with results from a Chinese study, where approximately two-thirds had less than high school education (Grady et al., 2017; Seo et al., 2022; Wang et al., 2021). Median income was low, with half (58%; CI 49%–66%) earning less than 20,000 SEK per month, as compared to the median employment income of 35,600 SEK in 2023 (Statistics Sweden, 2024a). Low income among healthy volunteers is well-described in existing literature (Almeida et al., 2007; Grady et al., 2017; Kalbaugh et al., 2021; Wang et al., 2021). All but one who reported an income of less than 10,000 SEK were students (who supposedly are living primarily on government student loans not considered income), unemployed or homemakers. Though most subjects reported low income, only one-third (32%; CI 24%–40%) reported being dissatisfied with their financial situation. This is comparable to a 2023 survey on the general population by the Swedish Financial Supervisory Authority and The Public Health Agency of Sweden, where one-third were dissatisfied with their current financial situation (The Swedish Financial Supervisory Authority, 2024). Only personal and not household income was collected in this study.

The observed unemployment rate (9%; CI 5%–15%) was unexpectedly lower than that reported in other studies, (Fisher et al., 2018; Grady et al., 2017; Kalbaugh et al., 2021; Wang et al., 2021) but comparable to the seasonally adjusted unemployment rate in the Swedish general population (8%, July 2024) (Statistics Sweden, 2024b). Unemployment did not appear to be a driver for participation per se. This could be attributed to the social welfare system in Sweden, where 72% of the working-age population were, in 2023, also members of a member-owned unemployment insurance fund (The Swedish Unemployment Insurance Inspectorate, 2024). This provides economic stability to unemployed individuals. Perhaps more importantly, financial compensation received from clinical trials is considered taxable income in Sweden. Any part-time income reduces unemployment benefits received, which diminishes the financial gain and incentive for unemployed individuals receiving benefits to participate in clinical trials. Based on our experience, compensation for Phase I trials in Sweden is typically determined by the wage-payment model (Dickert & Grady, 1999) without any completion bonuses. Compensation may range from around 10,000 SEK before taxes for shorter trials involving screening, a few days confinement, and a follow-up visit, to 20,000–30,000 SEK for trials with multiple or longer confinement periods, reflecting the time and burden on the volunteers.

The fact that most participants (73%; CI 65%–81%) had no children at home and half (50%; CI 42%–59%) were not employed, supports the assumption that having the time needed and flexible scheduling is a probable facilitator for participation (Manton et al., 2019). This was highlighted by some participants in their comments (Supplementary Material 4).

### Motivations, Trust and Social Influences

It is generally believed that healthy volunteers are primarily motivated by the financial compen­sation they receive (Almeida et al., 2007; Lemmens & Elliott, 1999). Our study found that while compensation is a significant motivator, other decision-influencing factors such as the competence and treatment from personnel, as well as regulatory review, followed by contributing to research, receiving a health check, and perceived low risk of side effects, were influential on decision-making. Several participants cited compensation as essential, but also noted concerns about side effects (Supplementary Material 4). This is consistent with existing literature. As far as we know, the largest survey among healthy volun­teers to date was conducted in the United States, Belgium, and Singapore between 2009 and 2011. This study found that perceived risk was the most important consideration while financial compensation was the primary motivation. It was even so concluded that multiple factors, including time commitment and quality of the facility and staff were taken into account (Grady et al., 2017). These conclusions have been confirmed in other studies and reviews, showing that a range of other concerns, including the possibility of contributing to research, learning about the scientific process, and study goals are considered by healthy volunteers (Fisher et al., 2018; Stunkel & Grady, 2011). Regional differences have also been found (Grady et al., 2017). For instance, a South Korean study concluded that various factors, including drug characteristics and frequency of visits, were influential on the decision to participate (Seo et al., 2022). A Saudi study on public motives determined that potential participants preferred studies of short duration, and studies conducted in a government hospital (Almutairi et al., 2019).

The high likelihoods of wanting to participate again (90%; CI 83%–94%) and recommending trial participation to friends or family (87%; CI 80%–92%), reflect the generally positive experiences of participants in our study. Reactions from friends and relatives were in contrast mixed (47% positive; CI 38%–56%), and one in four (26%; CI 19%–34%) had been advised against participating. In an Australian study, disapproval from family members was found to be a barrier, suggesting educational materials to relatives should be made available (Manton et al., 2019). Still, only 26% (CI 19%–34%) of participants in our study found the positive attitude from others to be important for their decision to participate. This suggests that the social validation from others played a minor role. Similar results have previously been reported, were many participated despite disapproving family members, or after having received recommendations not to do so by others (Almeida et al., 2007; Manton et al., 2019). Nonetheless, one participant expressed reluctance to discuss their participation with others due to not wanting to reveal financial difficulties (Supplementary Material 4). This suggests that there may among some be a stigma attached to volunteering for money. A general negative connotation of being a “guinea pig” has been described in other regions (Wang et al., 2021). Our experience is that such views are not commonly expressed in Sweden, though the perception that participating in clinical trials for money signals desperation could discourage individuals from discussing this openly.

Less than one in ten were likely to consider a trial with no financial compensation or no regulatory review (7% [CI 3%–12%] and 8% [CI 4%–14%], respectively). It is probable that participants do not consider these factors independently, and motivations may be influenced by the specifics of each trial. Results strongly support that competent and considerate site personnel, adequate regulatory review, and financial compensation are essential factors to healthy volunteers in Sweden.

The high levels of satisfaction with trial information, and trust in personnel and regulatory agencies, is not necessarily a measure of the quality of the trials or agencies, but rather reflect the significant role these factors play in volunteers’ decision-making processes. Individuals who felt unsure about the information given, or lacked trust in the personnel or regulatory oversight, may have chosen not to enroll in the first place, effectively self-selecting out. Similarly, the low level of concern about side effects in this study probably implies that individuals who are more worried are less likely to volunteer at all. Perceived importance of regulatory review and approval correlated with importance of low risk for side effects (r = 0.47, *p* < 0.001). Regulatory approval could consequently be considered a validation of the trial's safety and ethical standards. A noteworthy finding is that only one-third (34%; CI 26%–42%) were likely to participate in a trial reviewed solely by authorities in another EU country. It is possible participants may have concerns about differences in regulatory standards or prefer domestic institutions. The importance of regulatory approval to healthy volunteers might differ regionally depending on the general trust in public institutions. In other regions, greater emphasis may be put on other factors. For example, lack of trust in source of trial information and institutions has previously been described as an enrollment barrier for healthy volunteers in China (Wang et al., 2021).

### Preferences

Phase I trials in healthy volunteers range from exploratory trials and first-in-human safety trials to pharmacokinetic trials of well-known drugs. The variability in responses regarding willingness to join different trials shows that not all healthy volunteers are willing to enroll in all types of Phase I trials. For example, only about half (54%; CI 45%–62%) were likely willing to participate in a first-in-human trial. Participants who considered the low risk of side effects to be important (40%; CI 32%–49%) were less willing to enroll in a first-in-human trial (r = −0.44, *p* < 0.001) or a trial with serious drug reactions in animal studies (r = −0.40, *p* = 0.003). In pre-clinical toxicity studies, it is however generally relevant to find the maximum tolerated dose, in order to understand potential adverse effects (European Medicines Agency, 2013). Lack of adverse reactions in animals can in fact be undesirable because it may indicate insufficient testing. Educating participants about the purpose and design of pre-clinical studies, and how they are used to guide dose levels in clinical trials, could help alleviate some of these concerns.

A greater proportion reported being likely willing to enroll in a trial with expected mild side effects, as compared to a trial with an uncomfortable procedure (69% [CI 60%–76%] and 35% [CI 27%–44%], respectively). Unwillingness to participate in studies with invasive procedures has been previously described, (Chen et al., 2017) and our results reinforce the idea that burden and hardship not directly related to the possible risks or effects from the IMP is of importance for decision-making. This also extends to less obvious burdens, such as dissatisfaction with the food provided and privacy concerns related to living in close quarters with other participants, as mentioned by participants (Supplementary Material 4). Uncomfortable procedures might be necessary for safety monitoring purposes or for the objectives of the trial, but trial design should carefully consider the burdens to participants and whether any uncomfortable procedure can be ethically and scientifically justified.

Socio-demographic factors were not found to be associated with experiences, decision-influential factors, or preferences. Considerations and concerns were shared across demographic groups. This could reflect a degree of homogeneity in the population of healthy volunteers. Larger studies have similarly found that decision factors did not vary by e.g., income or employment (Grady et al., 2017).

### Limitations

While we did not find associations between the number of previous trials and other outcomes, there may still be unmeasured confounding factors related to specific trial or participant experiences that could have influenced responses. It is important to interpret hypothesis testing results with caution. Significance, and a lack thereof, does not prove that relationships do or do not exist. These patterns should be further explored in future studies and challenged in other regions.

We deliberately did not collect adverse event details (or any data that could be considered trial data) to avoid potential conflicts with GCP standards and to protect trial confidentiality. Collecting such data outside the trial could introduce unverified information and privacy risks. The absence of adverse event experiences limits the analysis, as it would have provided further insights into participants’ actual trial experiences and their impact on decision-making.

Additionally, the possibility of social desirability bias influencing responses cannot be ruled out, especially regarding questions about motivations for participation. This may have led some participants to underreport e.g., financial motivations. However, in our study, financial compensation was considered highly important, and few participants expressed willingness to participate in a trial without compensation.

Our study only reflects individuals who have already chosen to participate as a healthy volunteer, introducing opt-in bias. This study did not capture data on all participants at these research units, including volunteers who dropped out and did not return for their final visit. Though dropout rates in short-duration trials with healthy volunteers are generally low, it is possible that those who drop out would differ in their responses. Due to the proprietary nature of trial data and privacy constraints, we were unable to access or analyze information on total number of trial participants at these research units, or dropout rates. The results say little about those who do not volunteer for Phase I trials. Even if the results are generally in line with existing literature, the sample size might not be able to uncover subtle but meaningful patterns. The study is geographically limited to Sweden, and the findings should be generalized with caution to other regions where cultural, social, or economic factors might influence trial participation differently.

### Best Practices

Since healthy volunteers have no personal benefit from participation, there is already broad consensus that their safety and wellbeing are of utmost importance. Trial design should further prioritize minimizing both physical and psychological burdens. Financial compensation should be thoughtfully calibrated to ensure that volunteers are fairly compensated for their time and trouble, balancing the risk of exploitation (paying too little) with the risk of undue influence (paying too much). Participants in this study reported disproportionately low incomes but reported financial satisfaction comparable to the general population. There was no overrepresentation of migrants or unemployed individuals. Increasing compensation levels could broaden representation to higher-income groups, who may currently find the compensation insufficient to justify their participation. Trust in trial personnel and regulatory oversight also emerged as critical factors, supporting the ethical principle that trial personnel must always be mindful of the importance of transparent communication and respectful interactions with volunteers.

### Research Agenda

Further studies are necessary to generalize findings across different regions. More qualitative in-depth studies of healthy volunteers’ perceptions would provide valuable insights into their motivations, concerns, and experiences. Future research should include non-volunteers to better understand barriers to participation and ethical concerns. Societal views about participation in clinical research should be further researched.

### Educational Implications

Public engagement initiatives that provide accessible information about the clinical trial process, its benefits, risks, and burdens, could help build trust, enable informed decision-making, and increase diversity in Phase I trials. Training programs for investigators, sponsors, and ethics committees, should focus on the volunteer's perspective, with an emphasis on transparent and clear communication strategies. A better understanding of healthy volunteers’ motivations and vulnerabilities can help ethics committee members and policymakers in their review and oversight of clinical trials.

## Supplemental Material

sj-docx-1-jre-10.1177_15562646241309142 - Supplemental material for Characteristics, Motivations, and Preferences of Healthy Volunteers in Phase I Clinical Trials in Sweden Supplemental material, sj-docx-1-jre-10.1177_15562646241309142 for Characteristics, Motivations, and Preferences of Healthy Volunteers in Phase I Clinical Trials in Sweden by Erik Rein-Hedin, Mårten Schultzberg, Folke Sjöberg and Fredrik Huss in Journal of Empirical Research on Human Research Ethics

sj-docx-2-jre-10.1177_15562646241309142 - Supplemental material for Characteristics, Motivations, and Preferences of Healthy Volunteers in Phase I Clinical Trials in Sweden Supplemental material, sj-docx-2-jre-10.1177_15562646241309142 for Characteristics, Motivations, and Preferences of Healthy Volunteers in Phase I Clinical Trials in Sweden by Erik Rein-Hedin, Mårten Schultzberg, Folke Sjöberg and Fredrik Huss in Journal of Empirical Research on Human Research Ethics

sj-xlsx-3-jre-10.1177_15562646241309142 - Supplemental material for Characteristics, Motivations, and Preferences of Healthy Volunteers in Phase I Clinical Trials in Sweden Supplemental material, sj-xlsx-3-jre-10.1177_15562646241309142 for Characteristics, Motivations, and Preferences of Healthy Volunteers in Phase I Clinical Trials in Sweden by Erik Rein-Hedin, Mårten Schultzberg, Folke Sjöberg and Fredrik Huss in Journal of Empirical Research on Human Research Ethics

sj-docx-4-jre-10.1177_15562646241309142 - Supplemental material for Characteristics, Motivations, and Preferences of Healthy Volunteers in Phase I Clinical Trials in Sweden Supplemental material, sj-docx-4-jre-10.1177_15562646241309142 for Characteristics, Motivations, and Preferences of Healthy Volunteers in Phase I Clinical Trials in Sweden by Erik Rein-Hedin, Mårten Schultzberg, Folke Sjöberg and Fredrik Huss in Journal of Empirical Research on Human Research Ethics

sj-docx-5-jre-10.1177_15562646241309142 - Supplemental material for Characteristics, Motivations, and Preferences of Healthy Volunteers in Phase I Clinical Trials in Sweden Supplemental material, sj-docx-5-jre-10.1177_15562646241309142 for Characteristics, Motivations, and Preferences of Healthy Volunteers in Phase I Clinical Trials in Sweden by Erik Rein-Hedin, Mårten Schultzberg, Folke Sjöberg and Fredrik Huss in Journal of Empirical Research on Human Research Ethics

## References

[bibr1-15562646241309142] Almeida L. Azevedo B. Nunes T. Vaz-da-Silva M. Soares-da-Silva P. (2007). Why healthy subjects volunteer for Phase I studies and how they perceive their participation? European Journal of Clinical Pharmacology, 63(11), 1085–1094. 10.1007/s00228-007-0368-3 17891536

[bibr2-15562646241309142] Almutairi A. F. Almutairi B. M. Alturki A. S. Adlan A. A. Salam M. Al-Jeraisy M. I. Balkhy H. H. (2019). Public motives and willingness to participate in first-in-human clinical trials in Saudi Arabia: A new era in the making. Journal of Infection and Public Health, 12(5), 673–680. 10.1016/j.jiph.2019.03.013 31006634

[bibr3-15562646241309142] Best D. J. Roberts D. E. (1975). Algorithm AS 89: The upper tail probabilities of Spearman’s rho. Applied Statistics, 24(3), 377–379. 10.2307/2347111

[bibr4-15562646241309142] Bompart F. Fisher J. A. Allen E. Sevene E. Kumar N. Chew C. K. Fink V. Lanzerath D. Hirsch F. (2023). The VolREthics initiative to protect the well-being of healthy volunteers in biomedical research. Nature Medicine, 29(10), 2393–2394. 10.1038/s41591-023-02490-6 37580535

[bibr5-15562646241309142] Burgwal A. Gvianishvili N. Hård V. Kata J. García Nieto I. Orre C. Smiley A. Vidić J. Motmans J. (2019). Health disparities between binary and non binary trans people: A community-driven survey. International Journal of Transgenderism, 20(2–3), 218–229. 10.1080/15532739.2019.1629370 32999608 PMC6831016

[bibr6-15562646241309142] Chaudhry M. S. Spahn J. Patel S. Fuchs C. S. Lauchle J. Kolatkar N. Richie N. Highsmith Q. McKenzie M. Bhagat R. (2022). Myths about diversity in clinical trials reduce return on investment for industry. Nature Medicine, 28(8), 1520–1522. 10.1038/s41591-022-01858-4 35668180

[bibr7-15562646241309142] Chen S. C. Sinaii N. Bedarida G. Gregorio M. A. Emanuel E. Grady C. (2017). Phase 1 healthy volunteer willingness to participate and enrollment preferences. Clinical Trials, 14(5), 537–546. 10.1177/1740774517722131 28766409 PMC6010312

[bibr8-15562646241309142] Chen A. Wright H. Itana H. Elahi M. Igun A. Soon G. Pariser A. R. Fadiran E. O. (2018). Representation of women and minorities in clinical trials for new molecular entities and original therapeutic biologics approved by FDA CDER from 2013 to 2015. Journal of Women's Health, 27(4), 418–429. 10.1089/jwh.2016.6272 PMC7001461 29048983

[bibr9-15562646241309142] Clopper C. J. Pearson E. S. (1934). The use of confidence or fiducial limits illustrated in the case of the binomial. Biometrika, 26(4), 404–413. 10.1093/biomet/26.4.404

[bibr10-15562646241309142] CTTI. (n.d.). Patient group engagement. *CTTI*. Retrieved August 23, 2024, from https://ctti-clinicaltrials.org/our-work/patient-engagement/patients-groups-clinical-trials/

[bibr11-15562646241309142] Deiteren A. Coenen E. Lenders S. Verwilst P. Mannaert E. Rasschaert F. (2021). Data driven evaluation of healthy volunteer characteristics at screening for Phase I clinical trials to inform on study design and optimize screening processes. Clinical and Translational Science, 14(6), 2450–2460. 10.1111/cts.13113 34378856 PMC8604224

[bibr12-15562646241309142] Detoc M. Bruel S. Frappe P. Tardy B. Botelho-Nevers E. Gagneux-Brunon A. (2020). Intention to participate in a COVID-19 vaccine clinical trial and to get vaccinated against COVID-19 in France during the pandemic. Vaccine, 38(45), 7002–7006. 10.1016/j.vaccine.2020.09.041 32988688 PMC7498238

[bibr13-15562646241309142] Dickert N. Grady C. (1999). What’s the price of a research subject? Approaches to payment for research participation. New England Journal of Medicine, 341(3), 198–203. 10.1056/NEJM199907153410312 10403861

[bibr14-15562646241309142] Dinno A. (2024). dunn.test: Dunn’s Test of Multiple Comparisons Using Rank Sums (Version 1.3.6) [Computer software]. https://cran.r-project.org/web/packages/dunn.test

[bibr15-15562646241309142] Dresser R. (2009). First-in-human trial participants: Not a vulnerable population, but vulnerable nonetheless. The Journal of Law, Medicine & Ethics : A Journal of the American Society of Law, Medicine & Ethics, 37(1), 38–50. 10.1111/j.1748-720X.2009.00349.x PMC2692671 19245601

[bibr16-15562646241309142] Dunn O. J. (1964). Multiple comparisons using rank sums. Technometrics, 6(3), 241–252. 10.1080/00401706.1964.10490181

[bibr17-15562646241309142] EUPATI. (2023, July 18). *Guidance for patient involvement in regulatory processes*. EUPATI Toolbox. https://toolbox.eupati.eu/resources/patient-toolbox/guidance-for-patient-involvement-in-regulatory-processes/

[bibr18-15562646241309142] European Medicines Agency. (2013, February 11). ICH guideline M3(R2) on non-clinical safety studies for the conduct of human clinical trials and marketing authorisation for pharmaceuticals. https://www.ema.europa.eu/en/documents/scientific-guideline/ich-guideline-m3r2-non-clinical-safety-studies-conduct-human-clinical-trials-and-marketing-authorisation-pharmaceuticals-step-5_en.pdf

[bibr19-15562646241309142] European Medicines Agency. (2016, December 15). ICH guideline for good clinical practice E6(R2). https://www.ema.europa.eu/en/documents/scientific-guideline/ich-guideline-good-clinical-practice-e6r2-step-5_en.pdf

[bibr20-15562646241309142] Faulkner S. D. Somers F. Boudes M. Nafria B. Robinson P. (2023). Using patient perspectives to inform better clinical trial design and conduct: Current trends and future directions. Pharmaceutical Medicine, 37(2), 129–138. 10.1007/s40290-022-00458-4 36653601 PMC9848715

[bibr21-15562646241309142] Fisher J. A. McManus L. Wood M. M. Cottingham M. D. Kalbaugh J. M. Monahan T. Walker R. L. (2018). Healthy volunteers’ perceptions of the benefits of their participation in Phase I clinical trials. Journal of Empirical Research on Human Research Ethics, 13(5), 494–510. 10.1177/1556264618804962 30296882 PMC6235676

[bibr22-15562646241309142] U.S. Food and Drug Administration . (2019, April 18). *Payment and reimbursement to research subjects*. FDA. https://www.fda.gov/regulatory-information/search-fda-guidance-documents/payment-and-reimbursement-research-subjects

[bibr23-15562646241309142] U.S. Food and Drug Administration . (2024, March 7). *Diversity action plans to improve enrollment of participants from underrepresented populations in clinical studies*. FDA. https://www.fda.gov/regulatory-information/search-fda-guidance-documents/diversity-action-plans-improve-enrollment-participants-underrepresented-populations-clinical-studies

[bibr24-15562646241309142] German Federal Ministry of Education and Research. (n.d.). *Strengthening patient involvement in cancer research—BMBF*. Federal Ministry of Education and Research - BMBF. Retrieved August 7, 2024, from https://www.bmbf.de/bmbf/en/international-affairs/education-research-and-innovat–council-of-the-european-union/210907-unite-against-cancer.html

[bibr25-15562646241309142] Grady C. Bedarida G. Sinaii N. Gregorio M. A. Emanuel E. J. (2017). Motivations, enrollment decisions, and socio-demographic characteristics of healthy volunteers in phase 1 research. Clinical Trials (London, England), 14(5), 526–536. 10.1177/1740774517722130 28783972 PMC9896431

[bibr26-15562646241309142] Harrell F. E. J. (2024). Hmisc: Harrell Miscellaneous (Version 5.1-3) [Computer software]. https://CRAN.R-project.org/package=Hmisc

[bibr27-15562646241309142] Hochberg Y. (1988). A sharper Bonferroni procedure for multiple tests of significance. Biometrika, 75(4), 800–802. 10.1093/biomet/75.4.800

[bibr28-15562646241309142] Jain N. Cottingham M. D. Fisher J. A. (2020). Disadvantaged, outnumbered, and discouraged: Women’s experiences as healthy volunteers in U.S. Phase I trials. Critical Public Health, 30(2), 141–152. 10.1080/09581596.2018.1529861 32123487 PMC7050919

[bibr29-15562646241309142] Kalbaugh C. A. Kalbaugh J. M. McManus L. Fisher J. A. (2021). Healthy volunteers in US phase I clinical trials: Sociodemographic characteristics and participation over time. PLoS One, 16(9), e0256994. 10.1371/journal.pone.0256994 PMC8423261 34492044

[bibr30-15562646241309142] Kruskal W. H. Wallis W. A. (1952). Use of ranks in one-criterion variance analysis. Journal of the American Statistical Association, 47(260), 583–621. 10.2307/2280779

[bibr31-15562646241309142] Lamkin M. Elliott C. (2018). Avoiding exploitation in Phase I clinical trials: More than (un)just compensation. The Journal of Law, Medicine & Ethics, 46, 52–63. 10.1177/1073110518766008 PMC6047746 30026654

[bibr32-15562646241309142] Largent E. A. Fernandez Lynch H. (2017). Paying research participants: The outsized influence of “undue influence”. IRB, 39(4). https://pubmed.ncbi.nlm.nih.gov/29038611/ PMC5640154 29038611

[bibr33-15562646241309142] Largent E. A. Grady C. Miller F. G. Wertheimer A. (2012). Money, coercion, and undue inducement: Attitudes about payments to research participants. IRB, 34(1), 1–8. PMC4214066 22338401

[bibr34-15562646241309142] Lemmens T. Elliott C. (1999). Guinea Pigs on the payroll: The ethics of paying research subjects. Accountability in Research, 7(1), 3–20. 10.1080/08989629908573939 11657561

[bibr35-15562646241309142] Mann H. B. Whitney D. R. (1947). On a test of whether one of two random variables is stochastically larger than the other. The Annals of Mathematical Statistics, 18(1), 50–60. 10.1214/aoms/1177730491

[bibr36-15562646241309142] Manton K. J. Gauld C. S. White K. M. Griffin P. M. Elliott S. L. (2019). Qualitative study investigating the underlying motivations of healthy participants in Phase I clinical trials. BMJ Open, 9(1), e024224. 10.1136/bmjopen-2018-024224 PMC6340482 30647042

[bibr37-15562646241309142] May J. O. Looney S. W. (2020). Sample size charts for spearman and Kendall coefficients. Journal of Biometrics & Biostatistics, 11(2), 2. 10.37421/jbmbs.2020.11.440

[bibr38-15562646241309142] Regulation (EU) No 536/2014. (2022). https://eur-lex.europa.eu/eli/reg/2014/536/2022-12-05

[bibr39-15562646241309142] Seo J.-H. Kim O.-J. Yoo S.-H. Choi E. K. Park J.-E. (2022). A study on the characteristics of healthy volunteers who participate in Phase I clinical trials in Korea. Journal of Empirical Research on Human Research Ethics, 17(1–2), 193–212. 10.1177/15562646211034275 34414819

[bibr40-15562646241309142] Sharma N. S. (2015). Patient centric approach for clinical trials: Current trend and new opportunities. Perspectives in Clinical Research, 6(3), 134–138. 10.4103/2229-3485.159936 26229748 PMC4504054

[bibr41-15562646241309142] Statistics Sweden. (2024a, July 12). *Medianlöner i Sverige*. Statistikmyndigheten SCB. https://www.scb.se/hitta-statistik/sverige-i-siffror/utbildning-jobb-och-pengar/medianloner-i-sverige/

[bibr42-15562646241309142] Statistics Sweden. (2024b, August 23). *Arbetskraftsundersökningarna (AKU)*. Statistikmyndigheten SCB. https://www.scb.se/am0401

[bibr43-15562646241309142] Statistics Sweden. (n.d.). *Inrikes och utrikes födda efter region, ålder och kön. År 2000—2023*. Statistikdatabasen. Retrieved August 20, 2024, from http://www.statistikdatabasen.scb.se/pxweb/sv/ssd/START__BE__BE0101__BE0101E/InrUtrFoddaRegAlKon/

[bibr44-15562646241309142] Stunkel L. Grady C. (2011). More than the money: A review of the literature examining healthy volunteer motivations. Contemporary Clinical Trials, 32(3), 342–352. 10.1016/j.cct.2010.12.003 21146635 PMC4943215

[bibr45-15562646241309142] The Swedish Financial Supervisory Authority. (2024, January 17). *Ny undersökning: Ekonomisk ohälsa högre hos vissa grupper*. https://www.fi.se/sv/publicerat/rapporter/rapporter/2024/ny-undersokning-ekonomisk-ohalsa-hogre-hos-vissa-grupper/fi.se/sv/publicerat/rapporter/rapporter/2024/ny-undersokning-ekonomisk-ohalsa-hogre-hos-vissa-grupper/

[bibr46-15562646241309142] The Swedish Unemployment Insurance Inspectorate. (2024, February 23). *Sju av tio personer inom arbetskraften var med i en a-kassa 2023*. https://www.iaf.se/statistikdatabasen/arsstatistik/sju-av-tio-personer-inom-arbetskraften-var-med-i-en-a-kassa-2023/

[bibr47-15562646241309142] Treweek S. Pitkethly M. Cook J. Fraser C. Mitchell E. Sullivan F. Jackson C. Taskila T. K. Gardner H. (2018). Strategies to improve recruitment to randomised trials. Cochrane Database of Systematic Reviews, 2018(2), MR000013. 10.1002/14651858.MR000013.pub6 PMC7078793 29468635

[bibr48-15562646241309142] von Elm E. Altman D. G. Egger M. Pocock S. J. Gøtzsche P. C. Vandenbroucke J. P. , & STROBE Initiative. (2008). The Strengthening the Reporting of Observational Studies in Epidemiology (STROBE) statement: Guidelines for reporting observational studies. Journal of Clinical Epidemiology, 61(4), 344–349. 10.1016/j.jclinepi.2007.11.008 18313558

[bibr49-15562646241309142] Wang Z. Chen G. Liu X. Liu C. Song Q. Wang J. (2021). The motivations, barriers, and sociodemographic characteristics of healthy Chinese volunteers in Phase I research. European Journal of Clinical Pharmacology, 77(4), 557–568. 10.1007/s00228-020-03040-6 33188452

[bibr50-15562646241309142] Wickham H. Averick M. Bryan J. Chang W. McGowan L. D. François R. Grolemund G. Hayes A. Henry L. Hester J. Kuhn M. Pedersen T. L. Miller E. Bache S. M. Müller K. Ooms J. Robinson D. Seidel D. P. Spinu V. , … Yutani H. (2019). Welcome to the Tidyverse. Journal of Open Source Software, 4(43), 1686. 10.21105/joss.01686

