# Supplementary material for: Characteristics, Motivations, and Preferences of Healthy Volunteers in Phase I Clinical Trials in Sweden
Source: J Empir Res Hum Res Ethics. 2025 Jan 27;20(1-2):59–70. doi: 10.1177/15562646241309142 (PMC12052933; doi:10.1177/15562646241309142)
Supplement: sj-docx-1-jre-10.1177_15562646241309142 - Supplemental material for Characteristics, Motivations, and Preferences of Healthy Volunteers in Phase I Clinical Trials in Sweden [file sj-docx-1-jre-10.1177_15562646241309142.docx]

Survey for healthy volunteers who have participated in clinical trials
 **BACKGROUND***First, a few questions about yourself. Please select only one option unless otherwise indicated.*

| 1. Enter today’s date | year _______ | month _______ | day ________ |
| --- | --- | --- | --- |
| 1. How old are you? | years ______ |  |  |
|  |  |  |  |
| 1. What is your gender? | Female | Male | Other |

1. How would you describe your health?

|  | Very good |
| --- | --- |
|  | Fairly good |
|  | Neither good nor bad |
|  | Fairly bad |
|  | Very bad |

1. Where were you born?

|  | Sweden |
| --- | --- |
|  | Other country, which? ________________________ |

1. What is your current marital status?

|  | Single |
| --- | --- |
|  | Married |
|  | Domestic partnership |
|  | Living apart together |
|  | Divorced/separated |
|  | Widow/widower |

1. Do you have children living at home with you?

|  | Yes |
| --- | --- |
|  | No |

7b. If yes, what are the ages of the children? **(multiple choices possible)**

| 0-6 years | 7-12 years | 13-17 years | 18+ years |
| --- | --- | --- | --- |
|  |  |  |  |

1. What is your highest completed level of education?

|  | Less than 9 years of primary education |
| --- | --- |
|  | Primary education or equivalent |
|  | Upper secondary education or equivalent |
|  | Post-secondary non-tertiary education |
|  | Higher education at university/college level |
|  | University/college degree |

1. What is your main occupation?

|  | Employed |
| --- | --- |
|  | Unemployed |
|  | Parental leave |
|  | Homemaker |
|  | Student |
|  | Long-term sick leave/early retirement |
|  | Retired |

1. What is the travel distance from your home to the trial site?
   (Travel distance from Stockholm to Uppsala is approximately 70 km.)

| 0-9 km | 10-19 km | 20-49 km | 50-99 km | ≥ 100 km |
| --- | --- | --- | --- | --- |
|  |  |  |  |  |

1. What is your approximate gross income before taxes in a typical month?
   (Include any benefits, e.g. child allowance, housing allowance, study grants, etc.)

| 0-9 999 kr | 10-19 999 kr | 20-29 999 kr | 30-39 999 kr | 40-49 999 kr | 50-59 999 kr |
| --- | --- | --- | --- | --- | --- |
|  |  |  |  |  |  |
| 60-69 999 kr | 70-79 999 kr | 80-89 999 kr | 90-99 999 kr | ≥ 100 000 kr | Prefer not to disclose |
|  |  |  |  |  |  |

1. How satisfied are you overall with your financial situation?

|  | Very satisfied |
| --- | --- |
|  | Fairly satisfied |
|  | Neither satisfied nor dissatisfied |
|  | Fairly dissatisfied |
|  | Very dissatisfied |

**PREVIOUS EXPERIENCE***Here are questions about your experiences participating in trials. If you've been in multiple trials, focus primarily on the most recent trial you participated in. Please select only one option unless otherwise indicated.*

| 1. How many drug trials have you participated in? | 1 | 2 | 3 | 4 | ≥ 5 |
| --- | --- | --- | --- | --- | --- |
|  |  |  |  |  |  |
| 1. Is it likely that you would recommend a friend/family member to participate in a trial? | Very likely | Fairly likely | Neither likely nor unlikely | Not very likely | Not at all likely |
|  |  |  |  |  |  |
| 1. Before participating in the trial, did you discuss your participation with anyone? If yes, with whom? **(multiple choices possible)** | Yes, family member/partner | Yes, friend | Yes, healthcare professional | Yes, someone else | No |
|  |  |  |  |  |  |
| 1. What was the reaction of relatives and close friends to your decision to participate? | Very positive | Fairly positive | Neither positive nor negative | Fairly negative | Very negative |
|  |  |  |  |  |  |
| 1. What do you think of the financial compensation in relation to your participation? | More than sufficient | Sufficient | Neither sufficient nor insufficient | Not quite sufficient | Not at all sufficient |
|  |  |  |  |  |  |
| 1. What do you think of the oral and written information you received before participating in the trial? | More than sufficient | Sufficient | Neither sufficient nor insufficient | Not quite sufficient | Not at all sufficient |
|  |  |  |  |  |  |
| 1. During your participation, were you worried about experiencing side effects? | Very worried | Fairly worried | Slightly worried | Not very worried | Not at all worried |
|  |  |  |  |  |  |
| 1. How much trust do you have in the competence of the trial personnel (physicians and nurses) to conduct the trial? | Very high trust | Fairly high trust | Neither high nor low trust | Fairly low trust | Very low trust |
|  |  |  |  |  |  |
| 1. How much trust do you have in the Swedish authorities* that reviewed and approved the trial? *Swedish Medical Products Agency and Swedish Ethical Review Authority | Very high trust | Fairly high trust | Neither high nor low trust | Fairly low trust | Very low trust |
|  |  |  |  |  |  |

| 1. How challenging has participation been compared to your expectations? | Much more challenging | Slightly more challenging | As expected | Not as challenging | Not at all as challenging |
| --- | --- | --- | --- | --- | --- |
|  |  |  |  |  |  |
| 1. How likely are you to express interest in participating in more trials? | Very likely | Fairly likely | Neither likely nor unlikely | Not very likely | Not at all likely |
|  |  |  |  |  |  |

1. Has any family member/friend advised you against participating?

|  | Yes |
| --- | --- |
|  | No |

24b. If yes, what reasons have they given? **(multiple choices possible)**

| Missing time with family/social gatherings | Risk of side effects | Too low compensation | Other reason, specify |
| --- | --- | --- | --- |
|  |  |  | __________________________ |

**QUESTIONS ABOUT MOTIVATIONAL FACTORS**

*Now, questions about how important different factors are for your willingness to participate in a drug trial. Please select the option that fits best and only select one option per question.*

| 1. The trial being reviewed and approved by authorities | Very important | Fairly important | Neither important nor unimportant | Not very important | Not at all important |
| --- | --- | --- | --- | --- | --- |
|  |  |  |  |  |  |
| 1. Positive attitude from family and friends towards my participation | Very important | Fairly important | Neither important nor unimportant | Not very important | Not at all important |
|  |  |  |  |  |  |
| 1. Likelihood of experiencing side effects considered to be low | Very important | Fairly important | Neither important nor unimportant | Not very important | Not at all important |
|  |  |  |  |  |  |
| 1. Comprehensive health check-up as part of the trial | Very important | Fairly important | Neither important nor unimportant | Not very important | Not at all important |
|  |  |  |  |  |  |
| 1. Amount of financial compensation | Very important | Fairly important | Neither important nor unimportant | Not very important | Not at all important |
|  |  |  |  |  |  |
| 1. Competence of trial personnel (doctors and nurses) to conduct the trial | Very important | Fairly important | Neither important nor unimportant | Not very important | Not at all important |
|  |  |  |  |  |  |

| 1. Respectful/considerate treatment^*^ from trial personnel | Very important | Fairly important | Neither important nor unimportant | Not very important | Not at all important |
| --- | --- | --- | --- | --- | --- |
|  |  |  |  |  |  |
| 1. Drug indication (which medical condition it is intended to treat, e.g., diabetes, inflammation, depression, heart disease, etc.) | Very important | Fairly important | Neither important nor unimportant | Not very important | Not at all important |
|  |  |  |  |  |  |
| 1. How the drug is administered (tablet, solution, injection, cream, etc.) | Very important | Fairly important | Neither important nor unimportant | Not very important | Not at all important |
|  |  |  |  |  |  |

| 1. Contributing to medical research | Very important | Fairly important | Neither important nor unimportant | Not very important | Not at all important |
| --- | --- | --- | --- | --- | --- |
|  |  |  |  |  |  |
| 1. Opportunity to get to know and socialize with new people during the trial | Very important | Fairly important | Neither important nor unimportant | Not very important | Not at all important |
|  |  |  |  |  |  |
| 1. Learning about how clinical trials work | Very important | Fairly important | Neither important nor unimportant | Not very important | Not at all important |
|  |  |  |  |  |  |
| 1. Flexibility to change visit days and times to fit my life | Very important | Fairly important | Neither important nor unimportant | Not very important | Not at all important |
|  |  |  |  |  |  |
| 1. Short trial duration with few visits | Very important | Fairly important | Neither important nor unimportant | Not very important | Not at all important |
|  |  |  |  |  |  |
| 1. Standard of trial facilities (beds, access to common rooms, Wi-Fi, etc.) | Very important | Fairly important | Neither important nor unimportant | Not very important | Not at all important |
|  |  |  |  |  |  |

*Author comment on translation: In Swedish ”bemötande”, which can be translated as "treatment" or "interaction". It refers to how a person engages, treats, or acts towards others, and includes tone, attitude, and the way one deals with others. The importance of “bemötande” can be translated to the importance of respectful, attentive, and considerate treatment.

**WILLINGNESS TO PARTICIPATE IN DIFFERENT TYPES OF TRIALS**

*Now imagine that you are considering participating in a new trial. How likely would you be to consider participating in any of the following completely fictional trials? Please select the option that fits best and select only one option per question.*

| 1. A trial that has **not** been reviewed or approved by any authority (government agency) | Very likely | Fairly likely | Neither likely nor unlikely | Not very likely | Not at all likely |
| --- | --- | --- | --- | --- | --- |
|  |  |  |  |  |  |
| 1. A trial that has been reviewed and approved only by authorities in another EU country | Very likely | Fairly likely | Neither likely nor unlikely | Not very likely | Not at all likely |
|  |  |  |  |  |  |
| 1. A trial where you receive no financial compensation (only reimbursement for travel costs, etc.) | Very likely | Fairly likely | Neither likely nor unlikely | Not very likely | Not at all likely |
|  |  |  |  |  |  |
| 1. A trial where temporary, mild side effects can be expected (e.g. headache, nausea, fatigue) | Very likely | Fairly likely | Neither likely nor unlikely | Not very likely | Not at all likely |
|  |  |  |  |  |  |
| 1. A trial with an uncomfortable procedure (e.g. gastric tube insertion or cerebrospinal fluid sample) | Very likely | Fairly likely | Neither likely nor unlikely | Not very likely | Not at all likely |
|  |  |  |  |  |  |
| 1. A trial where you would need to be bedridden for parts of certain days due to frequent blood tests | Very likely | Fairly likely | Neither likely nor unlikely | Not very likely | Not at all likely |
|  |  |  |  |  |  |
| 1. A trial where serious side effects have been observed in animal studies at **much higher** doses than those used in the trial | Very likely | Fairly likely | Neither likely nor unlikely | Not very likely | Not at all likely |
|  |  |  |  |  |  |
| 1. A trial where the drug is a new vaccine | Very likely | Fairly likely | Neither likely nor unlikely | Not very likely | Not at all likely |
|  |  |  |  |  |  |
| 1. A trial where the medication is intended to affect the brain (e.g., medication for depression and neurological diseases) | Very likely | Fairly likely | Neither likely nor unlikely | Not very likely | Not at all likely |
|  |  |  |  |  |  |
| 1. A trial with long visits where you need to stay many nights | Very likely | Fairly likely | Neither likely nor unlikely | Not very likely | Not at all likely |
|  |  |  |  |  |  |
| 1. A trial with frequent visits requiring many trips to and from the clinic (travel expenses reimbursed) | Very likely | Fairly likely | Neither likely nor unlikely | Not very likely | Not at all likely |
|  |  |  |  |  |  |
| 1. A trial conducted at a clinic far from my home (over 1 hour travel time) | Very likely | Fairly likely | Neither likely nor unlikely | Not very likely | Not at all likely |
|  |  |  |  |  |  |
| 1. A trial with a drug being tested for the very first time in humans | Very likely | Fairly likely | Neither likely nor unlikely | Not very likely | Not at all likely |
|  |  |  |  |  |  |
| 1. A trial where the sponsor is a large, foreign, well-known company | Very likely | Fairly likely | Neither likely nor unlikely | Not very likely | Not at all likely |
|  |  |  |  |  |  |
| 1. A trial where the sponsor is a small, unknown, Swedish company | Very likely | Fairly likely | Neither likely nor unlikely | Not very likely | Not at all likely |
|  |  |  |  |  |  |

1. Additional comments

______________________________________________________________________________________________________________________________________________________________________________________
___________________________________________________________________________________________
 **THANK YOU VERY MUCH FOR YOUR PARTICIPATION!**
