# Supplementary material for: Characteristics, Motivations, and Preferences of Healthy Volunteers in Phase I Clinical Trials in Sweden
Source: J Empir Res Hum Res Ethics. 2025 Jan 27;20(1-2):59–70. doi: 10.1177/15562646241309142 (PMC12052933; doi:10.1177/15562646241309142)
Supplement: sj-docx-2-jre-10.1177_15562646241309142 - Supplemental material for Characteristics, Motivations, and Preferences of Healthy Volunteers in Phase I Clinical Trials in Sweden [file sj-docx-2-jre-10.1177_15562646241309142.docx]

**Suppl Figure 1.** Correlation Heatmap. Heatmap displays significant Spearman correlations (p < 0.05) between variables after Hochberg correction
for multiple comparisons. Strength and direction of correlations are represented by color gradients. Numbers correspond to variables provided in separate legend.


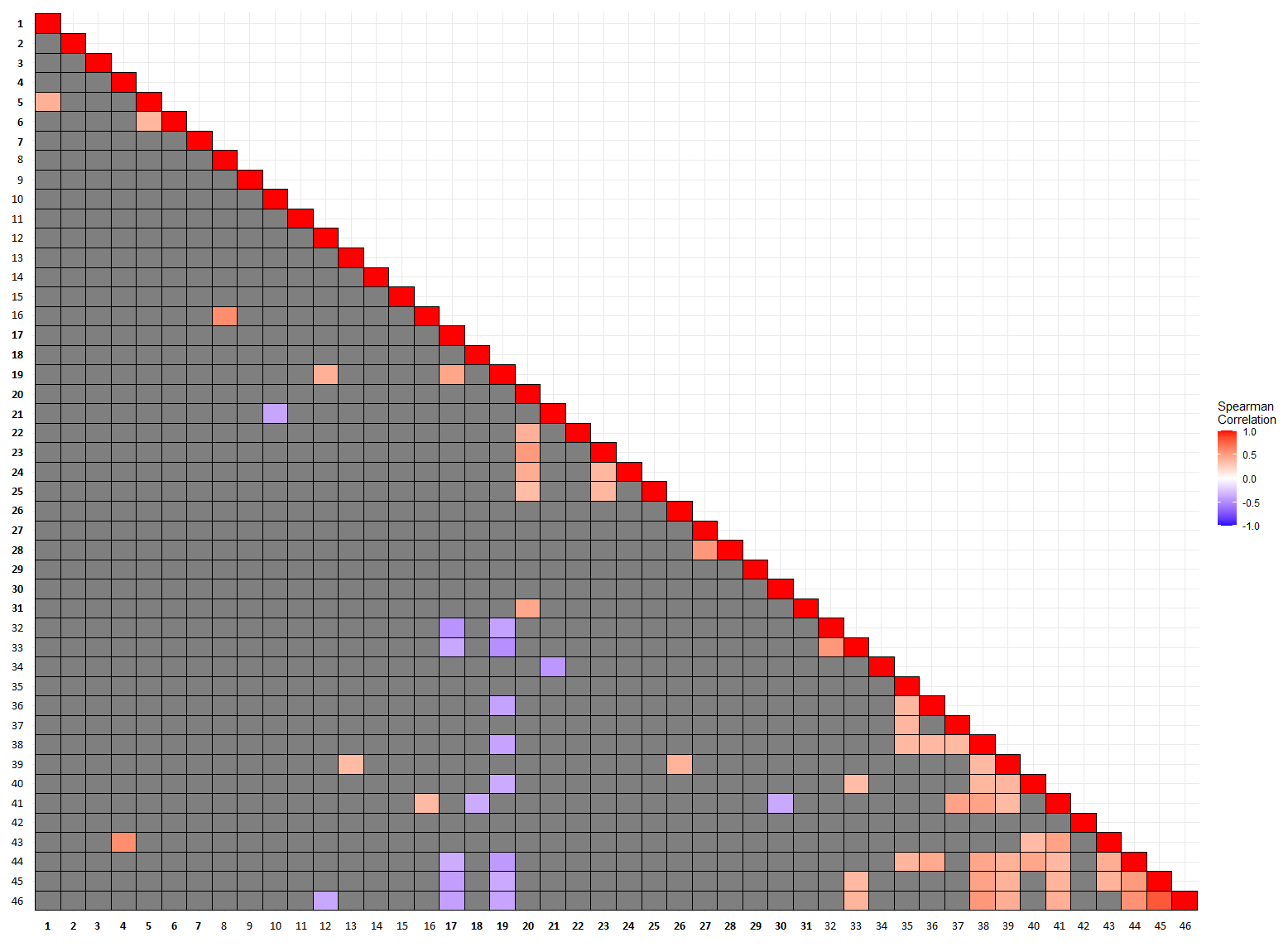


| **Background characteristics** | |  | 23 | Respectful/considerate treatment from trial personnel |
| --- | --- | --- | --- | --- |
| 1 | Age |  | 24 | Drug indication |
| 2 | Perceived health |  | 25 | Drug administration method |
| 3 | Education level |  | 26 | Contributing to medical research |
| 4 | Distance to trial site |  | 27 | Opportunity to get to know and socialize with new people |
| 5 | Income |  | 28 | Learning about clinical trials |
| 6 | Financial satisfaction |  | 29 | Flexible trial schedules |
| 7 | Previous number of trials |  | 30 | Short trial duration |
| **Experiences** | |  | 31 | Standard of trial facilities |
| 8 | Likely to recommend participation |  | **Willingness to participate in different types of trials** | |
| 9 | Positive reactions from others |  | 32 | A trial with no regulatory review or approval |
| 10 | Consider financial compensation sufficient |  | 33 | A trial only reviewed and approved by another EU country |
| 11 | Consider trial information sufficient |  | 34 | A trial with no financial compensation |
| 12 | Worried about side effects |  | 35 | A trial with expected mild side effects |
| 13 | Trust in competence of trial personnel |  | 36 | A trial with an uncomfortable procedure |
| 14 | Trust in regulatory agencies* |  | 37 | A trial with bedridden periods |
| 15 | Participation challenging compared to expectations |  | 38 | A trial with serious side effects in animal studies at much higher doses |
| 16 | Likely to participate in future trials |  | 39 | A trial with a new vaccine |
| **Perceived importance of factors** | |  | 40 | A trial with a drug intended to affect the brain |
| 17 | Regulatory review and approval |  | 41 | A trial with many nights at the clinic |
| 18 | Positive attitude from family and friends |  | 42 | A trial with frequent visits |
| 19 | Low probability of side effects |  | 43 | A trial conducted far from my home |
| 20 | Receiving a comprehensive health check-up |  | 44 | A first-in-human trial |
| 21 | Amount of financial compensation |  | 45 | A trial with a large, foreign, well-known sponsor |
| 22 | Competence of trial personnel |  | 46 | A trial with a small, unkown, Swedish sponsor |

* Swedish Medical products Agency and Swedish Ethical Review Authority

**Legend Suppl Figure 1.** Numeric labels for variables.
