# Supplementary material for: Characteristics, Motivations, and Preferences of Healthy Volunteers in Phase I Clinical Trials in Sweden
Source: J Empir Res Hum Res Ethics. 2025 Jan 27;20(1-2):59–70. doi: 10.1177/15562646241309142 (PMC12052933; doi:10.1177/15562646241309142)
Supplement: sj-docx-4-jre-10.1177_15562646241309142 - Supplemental material for Characteristics, Motivations, and Preferences of Healthy Volunteers in Phase I Clinical Trials in Sweden [file sj-docx-4-jre-10.1177_15562646241309142.docx]

Comments and feedback

Translated from Swedish to English. Personal identifiable information has been redacted. Comments are unfiltered and all feedback is included.

| 1 | For me, the consideration of whether I want to participate in a trial is above all about the compensation and potential side effects. I don’t want to be exposed to side effects that could be long-lasting or affect my everyday life, and I need to feel that the compensation is reasonable in relation to both the time spent and any potential discomfort or pain that might be involved. |
| --- | --- |
| 2 | Being even more forewarned about possible side effects would have been helpful. I became worried and surprised. |
| 3 | Taking the survey, you should have the option to choose ”never in a million years” as your answer to the questions. The compensation is way too low since a lot is lost due to taxes and tax deductions for meals provided. It’s also important to speed up payment so that you don’t have to wait for too long. I would like access to more TVs and extra computers. Food portions could be larger. More chicken and beef, please. Raw sugar instead of white sugar. |
| 4 | Thanks to the super professional staff. Especially, who is able to be professional and clear at the same time as being interested, open and cheerful. |
| 5 | Thanks for your pleasant and kind treatment and all the great conversations that raised our spirits while you were working with the participants. Especially, who seems to love their job. Very good with nice and cozy facilities that are well-equipped and much more pleasant than the hospital environment, which is too gloomy, depressing, dull, and sterile. |
| 6 | From question 40 and onwards it wasn’t entirely easy to stay focused on the questions and provide accurate answers. |
| 7 | There should be more space between the questions. |
| 8 | I think what would attract more people is higher compensation. |
| 9 | I mainly participated in my trial because the clinic is nearby, it fit with my schedule, and the research is relevant to me. |
| 10 | Another important factor for me is the opportunity to follow the study and investigational drug, its results, or implications, out of curiosity and interest. If that’s a possibility! |
| 11 | For question 53 [“How likely would you be to consider participating in a trial where the sponsor is a large, foreign, well-known company?”], it depends on the company’s reputation. For question 51 [“How likely would you be to consider participating in a trial conducted at a clinic far from your home?”], I can consider traveling more than an hour if the compensation is high and I can stay there for several nights instead of having many return visits! |
| 12 | Thanks for great service and good treatment! |
| 13 | The absolute most important factor is the amount of compensation. That always determines whether I want to participate or not. |
| 14 | I consider the compensation in relation to time spent, risk, and overall discomfort, also considering the possibility of participating in other trials. This is what is most important to me. |
| 15 | Prefer longer stays (preferably in one stretch) meaning higher (and easier) compensation. Traveling back and forth for day visits is more work. |
| 16 | Side effects that you experience yourself aren’t as scary as side effects that could cause birth defects. |
| 17 | It’s hard to generalize. Everything is related. It’s a balance between value, demands, and compensation. |
| 18 | I would say that my participation was primarily motivated by financial reasons, but now that I’ve been part of a trial, I’ve discovered other benefits, like it being a really interesting experience! |
| 19 | The most important thing for me is the financial compensation and the trial’s "safety”. Those are the variables that make a trial interesting for me to participate in or not. |
| 20 | Everything depends on the compensation, even though it’s more likely that you participate in something that personally affects you. I don’t tell others [that I am a research participant] because I don’t want them to know that I have financial difficulties. |
| 21 | We were supposed to get fruit as a snack, according to the meal plan, but we didn’t get any. |
| 22 | A big impact on my decision is how the compensation compares to loss of income if I need to take time off work. |
| 23 | It would be great to have more toilets, especially in a more private location. It’s unpleasant to have to use the bathroom in front of other participants (I mean the bathroom is in the hallway where everyone passes by). It’s noisy and a bit stressful because others might also need the toilet (waiting in line). I’d prefer a calmer situation for using the bathroom. Big thanks to the amazing staff! |
| 24 | I don’t think the bed mattresses have been changed since 2019 when I did my first trial. That, and the quality of the pillow are my main complaints. It could have been a greater variety of meals served. That said, it was still super nice to choose lunch from a nearby restaurant on some days! A real morale booster! |
| 25 | It’s mainly the compensation that motivates me, but a plus is contributing to research. The higher the compensation, the worse/more I can be subjected to. |
| 26 | Since I had side effects, more information about them would have been good. Despite that, I still had a very positive experience with the trial. |
| 27 | I prefer a longer overnight stay at the clinic over many short visits. This time, the atmosphere was pretty stressful, both among us participants and the staff. |
| 28 | The most important thing for me is that the compensation is sufficient, the perceived risk is relatively low, and that I can do the trial without it interfering with important things in life. |
